# An efficient graph theory based method to identify every minimal reaction set in a metabolic network

**DOI:** 10.1186/1752-0509-8-28

**Published:** 2014-03-04

**Authors:** Sudhakar Jonnalagadda, Rajagopalan Srinivasan

**Affiliations:** 1 Institute of Chemical and Engineering Sciences, Agency for Science, Technology and Research (A*STAR), 1 Pesek Road, Jurong Island 627833, Singapore; 2 Department of Chemical and Biomolecular Engineering, National University of Singapore, 10 Kent Ridge Crescent 119260, Singapore; 3 Current Affiliation: Indian Institute of Technology Gandhinagar, Vishwakarma Government Engineering College Complex, Chandkheda, Ahmedabad, Gujarat, Gandhinagar 382424, India

**Keywords:** Systems biotechnology, Strain development, Minimal cell, Mixed-Integer Linear Program (MILP), Multiple solutions

## Abstract

**Background:**

Development of cells with minimal metabolic functionality is gaining importance due to their efficiency in producing chemicals and fuels. Existing computational methods to identify minimal reaction sets in metabolic networks are computationally expensive. Further, they identify only one of the several possible minimal reaction sets.

**Results:**

In this paper, we propose an efficient graph theory based recursive optimization approach to identify all minimal reaction sets. Graph theoretical insights offer systematic methods to not only reduce the number of variables in math programming and increase its computational efficiency, but also provide efficient ways to find multiple optimal solutions. The efficacy of the proposed approach is demonstrated using case studies from *Escherichia coli* and *Saccharomyces cerevisiae*. In case study 1, the proposed method identified three minimal reaction sets each containing 38 reactions in *Escherichia coli* central metabolic network with 77 reactions. Analysis of these three minimal reaction sets revealed that one of them is more suitable for developing minimal metabolism cell compared to other two due to practically achievable internal flux distribution. In case study 2, the proposed method identified 256 minimal reaction sets from the *Saccharomyces cerevisiae* genome scale metabolic network with 620 reactions. The proposed method required only 4.5 hours to identify all the 256 minimal reaction sets and has shown a significant reduction (approximately 80%) in the solution time when compared to the existing methods for finding minimal reaction set.

**Conclusions:**

Identification of all minimal reactions sets in metabolic networks is essential since different minimal reaction sets have different properties that effect the bioprocess development. The proposed method correctly identified all minimal reaction sets in a both the case studies. The proposed method is computationally efficient compared to other methods for finding minimal reaction sets and useful to employ with genome-scale metabolic networks.

## Background

The depletion of fossil fuels and increasing concerns over environmental changes are key driving factors for the development of sustainable bioprocesses to produce chemicals and fuels from renewable resources [1]. Today, bioprocesses using microorganisms are being increasingly used for production of compounds with applications in food, agriculture, chemical and pharmaceutical industries [2-4]. Bioprocesses provide several advantages over traditional chemical processes including high specificity, low temperature, low pressure and reduced use of strong solvents; thus they are environmentally friendlier while reducing the dependency on fossil resources. Despite these advantages, the industry has not adapted bioprocesses extensively, because the viability of bioprocesses is often questionable due to low yield and productivity for desired compounds [5]. In order to make bioprocesses economically viable, it is essential to engineer microbial strains that offer enhanced yield of the desired product [6,7].

Synthetic biology provides the tools and techniques to design and construct artificial cells with minimal functionality containing a minimal genome, but with all the essential genes for survival in a defined environment and possessing replication capabilities [8]. Such minimal cells provide a platform for efficient production of desired chemicals and decontamination of waste streams [9,10]. Strains with reduced genomes have been created by deleting large number of non-essential genes [11,12]. These strains have shown to have equal or better growth performance compared to their parent strains [13,14]. In biotechnology applications, improved performance has been reported by the strains with minimal metabolism created by blocking handful of reactions that drive the metabolic flux through the predefined minimal metabolic reactions [15]. Burgard et al. [16] proposed a mathematical programming approach to find the minimal reaction sets under different uptake environments. Their study finds that minimal reaction sets are strongly dependent on medium constituents and cellular objectives. This approach does not provide any indication on what reactions have to be blocked in order to construct the cell with minimal metabolism besides its computational complexity is high. The approach used by Trinh el at. [15] identifies the reactions to be blocked to design the cell with minimal metabolism by considering the reduction in Elementary Flux Modes (EFMs) achieved by removing reactions from the metabolic model. EFMs represent the various independent pathways available for the cell to achieve its cellular objectives [17]. EFMs analysis has so far been employed only with small metabolic models representing the central metabolism but the computational complexity of EFMs analysis prevents its application to genome-scale models. We have previously proposed a graph theory based approach for identifying minimal reaction set in metabolic networks [18]. The approach exploits the network structure of metabolic networks and uses math programming efficiently, to identify the minimal reaction set. Significant reduction in the computational time has been achieved using the graph theory based approach compared to classical math programming.

The presence of redundant pathways in metabolic networks results in alternate optimal solutions and consequently create mismatch between model predictions and experimental observations [19,20]. Redundant pathways also lead to multiple minimal reaction sets with different biological significance. Several factors related to the physical and biological functioning of the designed cells including high substrate utilization, deregulated pathways, high tolerance to inhibitors, robust reproduction, predictable metabolic interactions, and physical robustness to sustain the stress and strain during fermentation have to be considered before creating minimal cells [21,22]. Though not all these factors may be equally important in designing minimal metabolic cells, some such as practically achievable metabolic fluxes, thermodynamically favourable pathways, and high substrate utilization should be incorporated. Also, the number of reactions to be knocked-out in order to create a minimal metabolism cell is an important factor. Each solution, minimal metabolism cell, found through computational analysis of metabolic network has different properties that may not be captured in the model used for computational analysis. Identifying all the minimal reaction sets would enable us to evaluate such non-quantifiable properties of different minimal reaction sets and select the one most suitable for experimental development. In this paper, we propose a graph theory based recursive math programming approach to identify all the minimal reaction sets in the metabolic network.

## Methods

### MILP for finding minimal reaction set

The metabolic network of a given microorganism with *N* metabolites and *M* reactions is mathematically represented as [23]:

(1) $$\sum_{j=1}^{M}S_{\mathrm{ij}}v_{j}=0,\;i=1,2,\ldots ,N$$

The Stoichiometric matrix *S* captures interactions among reactions where *S*_*ij*_ is the stoichiometric coefficient of the *i*^*th*^ metabolite in the *j*^*th*^ reaction and *v*_*j*_ is the flux (rate) of reaction *j*. The zero in the right hand side is due to the steady-state assumption generally considered in metabolic network analysis. The mathematical representation of metabolic networks enables analysis of the metabolism using optimization methods to identify internal flux distribution, metabolic capabilities, and strain improvement strategies through gene knock-out or insertion of non-native reactions [17,24-26]. Identification of minimal reaction set can be represented as an optimization problem given by [16]:

(2) $$\begin{array}{l} \text{minimize}\;z=\sum_{j=1}^{M}y_{j}\\ s.t\;\sum_{j=1}^{M}S_{\mathrm{ij}}v_{j}=0\;i=1,2,\ldots ,N\\ v_{j}^{\text{min}}\cdot y_{j}\leq v_{j}\leq v_{j}^{\text{max}}\cdot y_{j}\;j=1,2,\ldots ,M\\ y_{j}\in \left\{0,1\right\}\;j=1,2,\ldots ,M\\ v_{\mathrm{biomass}}\geq v_{\mathrm{biomass}}^{\text{max}}v_{j}\in R \end{array}$$

Here, *v*_*j*_^*min*^ and *v*_*j*_^*max*^ represent the lower and upper bounds for the flux through reaction *j*. A binary variable *y*_*j*_ is associated with each reaction with ‘1’ indicating the presence/activation of the reaction and ‘0’ its absence/deactivation. Cellular objectives are incorporated as constraints, for example, the objective in Eq. (2) is to produce at least $\nu \begin{array}{c} \max\\ \mathrm{biomass} \end{array}$ biomass. Although the Mixed-Integer Linear Programming (MILP) in Eq. (2) has been reported to be successfully solved in some cases, the computational time increases exponentially with number of reactions [18].

**Figure 1:**
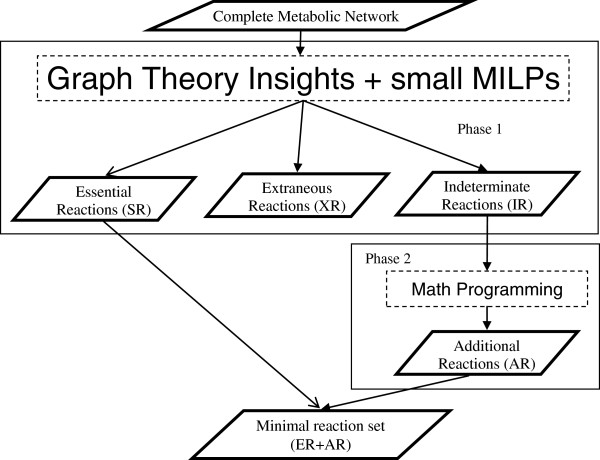
Schematic representation of the hybrid approach that combines graph theory insights with math programming to identify minimal reaction set.

We previously reported an efficient approach that combines graph theory with math programming to solve this problem (Jonnalagadda et al. [18]). In our hybrid approach, a metabolic network is considered as an AND-OR graph where nodes represent metabolites and arcs represent reactions. Reactions that require multiple metabolites to proceed are considered to be related by a AND-logic, while reactions that can produce or consume a metabolite using independent routes are considered to be conjoined by an OR logic. A *depth* is associated with each node and arc in the network starting with the extracellular metabolites and primary uptake reactions which are deemed to be of depth 1. The depth of every other metabolite and reaction is assigned as an increment over its predecessor’s. There are two phases in the hybrid approach (Figure 1). Based on the *depth* of reactions, Phase 1 decomposes the metabolic network into sub-networks which are then analyzed in isolation using small MILPs to classify reactions as Essential, Extraneous or Indeterminate. *Essential reactions* (SRs) are required for the cell to meet biological objectives and hence they are the part of every minimal set. *Extraneous* reactions (XRs) are not necessary for the cell. *Indeterminate reactions* (IRs) primarily consist of substitutable reactions *i.e. reactions which can be substituted with other reactions to achieve the cellular objectives.* These IRs are holistically analysed in the subsequent Phase 2, using a MILP with the same structure as that in (2) but smaller than the monolithic one. Through this, a subset of IRs called Additional reactions (ARs) necessary for the minimal metabolism cell are identified which together with SRs identified in Phase 1 form the minimal reaction set. A substantial reduction in the computational time (~66%) required to identify one solution could be achieved through the hybrid approach compared to solving Eq. (2) directly. In this paper, we extend the above hybrid approach to identify *all* minimal reaction sets. The theoretical basis of the proposed approach is discussed first.

#### Reaction dependency and grouping

**Figure 2:**
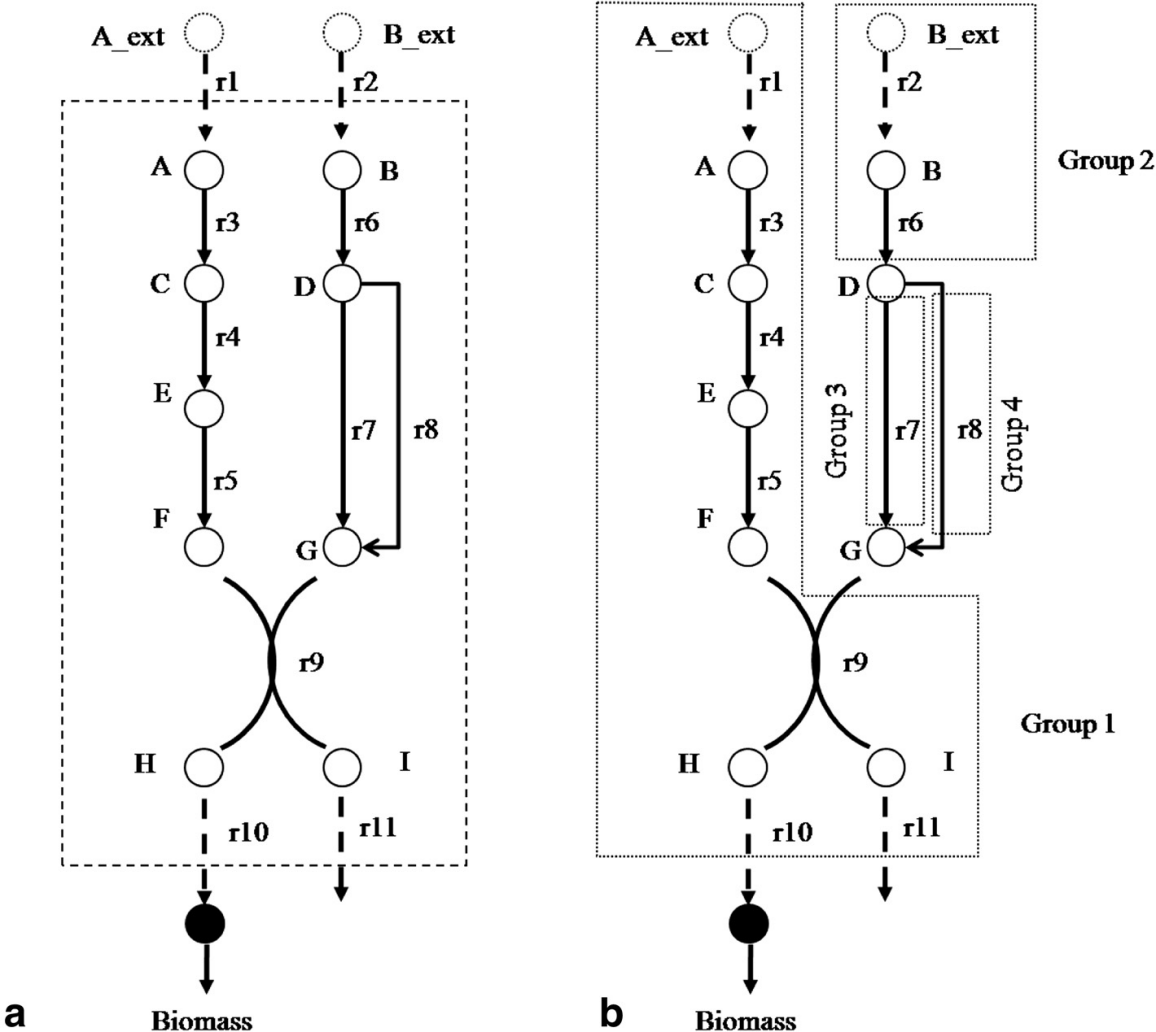
Sample metabolic network and groups of dependent reactions (a) A sample metabolic network (b) Four groups of dependent reactions in the network.

Reactions in the metabolic network are dependent on each other since the network is an interconnected system designed to achieve the biological objectives of the cell. Two different kinds of dependencies can be identified – *linear* and *flux dependency.* Linear dependency arises between reactions due to the structure of the network where the product(s) of a reaction feed into exactly one other reaction. When a set of reactions are all linearly dependent, they can be considered to form a linear pathway. Instances of several reactions forming a linear pathway in metabolic networks are common. For example, in the sample metabolic network shown in Figure 2a, two external metabolites *A_ext* and *B_ext* enter into the cell and biomass is produced from them through the reaction network. Two linear pathways can be identified in this sample network *{r*_*1,*_*r*_*3*_*, r*_*4,*_*r*_*5*_*}* and *{r*_*2,*_*r*_*6*_*}*. In linear pathways, under steady-state assumption mentioned above, the flux through all the reactions has to be equal. Hence, deletion of any reaction in the linear pathway would result in the deletion of the whole pathway.

Another kind of dependency, flux dependency, exists between reactions which are not structurally linear, but are required to co-exist to balance the fluxes. If a reaction produces two products which are then consumed by two different reactions, then these three reactions are dependent on each other, since at steady state, the metabolites produced by the first reaction have to be consumed by the two down-stream reactions. Deletion of any one of these reactions would make all other reactions incapable of carrying flux at steady state. In the sample network, although reactions *{r*_*9*_*, r*_*10*_*, r*_*11*_*}* are not linearly dependent, they have flux dependencies, since reactions *{r*_*10*_*, r*_*11*_*}* consume the products of *r*_*9*_ and are dependent on each other through the flux balance requirement.

The linear and flux dependency among reactions in a metabolic network can be exploited to assemble reactions into groups for analysis, rather than analyzing them individually. Since deletion of any reaction would force all the dependent reactions to be excluded from the network, for network optimization using a MILP, it is sufficient to associate a single binary variable with each group of reactions. Reduction of the number of binary variables reduces the search space and consequently reduces the computational cost of finding solutions. Identification of dependent reactions and simplification of metabolic networks using the reaction dependency has been reported in literature ([17,27,28]). Groups of dependent reactions are generally identified by comparing the rows in the null-space matrix of the Stoichiometric matrix, *S*. The null-space represents all the possible steady-state flux distributions that satisfy Eq. 1 and the dependent reactions are the rows in this matrix with same values after normalization with no contradictions in the directionality for irreversible reactions. Since this procedure depends on the directionality of reactions without considering the structural features of metabolic netwotk, it may not identify some dependent reaction groups due to imperfect assignment of reaction directionality in the metabolic networks. Also, identification of dependent reaction groups strictly based on flux distributions results in groups of structurally unrelated reactions which hinders interpretation. We have developed a graph based algorithm that exploits the structure of metabolic network to identify groups of dependent reactions as described next.

Given a metabolic network where the depth has been assigned to reactions as described in MILP for finding minimal reaction set, the algorithm first creates a list of reactions, sorted in the ascending order of their depth. Reactions are grouped from this list using an iterative procedure. In each iteration, a new group is created starting with the first reaction in the list, *i.e. reaction with the lowest depth*. Dependent reactions are then added to this group step-by-step by searching the metabolic network in a breath-first manner. In each step, all the reactions at the current depth +1 are collected and tested for linear or flux dependency. Reactions are added to the group if they dependent on other reactions that are already present in the group. Specifically, a single reaction that receives its reactants exclusively from another reaction in that group is deemed as linearly dependent. Similarly, multiple reactions are deemed to have flux dependency if all their reactants originate from one reaction already in the group. The search continues until al the reactions are evaluated i.e., the highest depth in the network is reached or no dependent reaction can be found at a given depth. Once a group of dependent reactions is identified, all these reactions are removed from the reaction list. In the subsequent iteration, the algorithm continues with the creation of a new group with the first reaction in the updated reaction list. The algorithm terminates when the reaction list becomes empty.

We illustrate the algorithm using the sample network shown in Figure 2a. In the first iteration, the algorithm starts a new group with reaction *r*_*1*_ (depth 1), identifies reaction *r*_*3*_ as linearly dependent at depth 2 in the first step, and then reaction *r*_*4*_ in the second step, and *r*_*5*_ in the third step. In the fourth step, *r*_*9*_ is added to the group since one of its two reactants is exclusively from *r*_*5*_. Reactions *{r*_*10,*_*r*_*11*_*}* are then identified as having flux dependency since they exclusively receive their reactants from *r*_*9*_. The search stops at this step since there are no further reactions in the list at higher depths. Thus the first group of dependent reactions is *{r*_*1,*_*r*_*3*_*, r*_*4,*_*r*_*5,*_*r*_*9,*_*r*_*10,*_*r*_*11*_*}*. The graph based algorithm thus groups reactions based on both linear and flux dependencies. The number of reactions in a group is called its norm. Thus, the norm of this group is 7. All these 7 reactions are removed from the reaction list. The second iteration starts with reaction *r*_*2*_ (since it has the lowest depth of the reactions in the updated reaction list), and identifies reaction *r*_*6*_ as its dependent. The search for dependent reactions stops here since two reactions, *{r*_*7*_*, r*_*8*_*}*, consume the product (D) of *r*_*6*_. Hence, the second group has 2 reactions *{r*_*2,*_*r*_*6*_*}*. Continuing in this fashion, two single reaction groups *{r*_*7*_*}* and *{r*_*8*_*}* are also identified. Thus, in total, there are four different groups in the sample network as shown in Figure 2b.

Once the groups of dependent reactions have been identified in the metabolic network, analysis can be carried out on these groups rather than on the individual reactions. If a reaction from a group is essential for the cell, all the reactions in that group become essential since they are dependent on each other. Similarly, all the reactions in the group become extraneous or indeterminate if one of the reactions in the group is extraneous or indeterminate, respectively. Hence, the minimal reaction set identification problem is reduced to identification essential reaction groups (SRGs), extraneous reaction groups (XRGs), and indeterminate reaction groups (IRGs).

#### Recursive MILP for finding all minimal reaction sets

**Figure 3:**
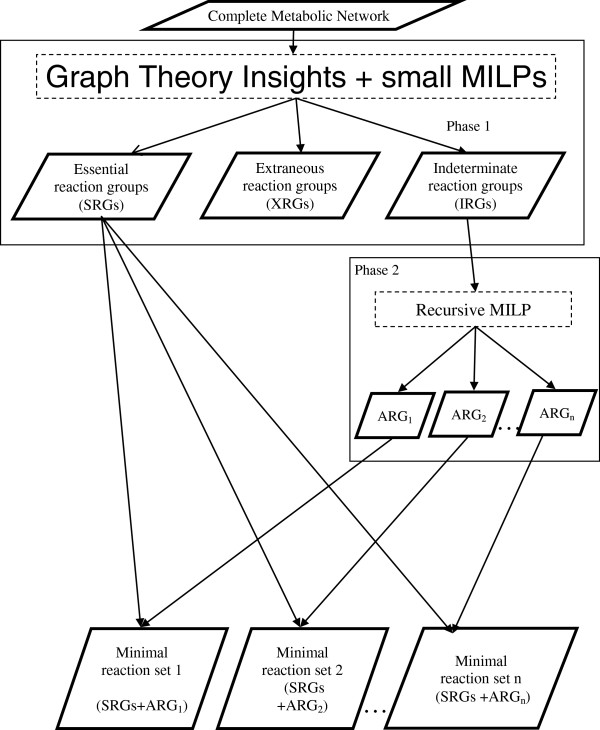
Schematic representation of the recursive MILP approach for identifying all minimal reaction sets.

The proposed recursive MILP approach for identifying all the minimal reaction sets in metabolic network is shown in Figure 3. A given metabolic network is described using the groups of dependent reactions where a single binary variable is associated with each group. Then, Phase 1 of the proposed approach classifies these groups into essential, extraneous and indeterminate groups using the algorithm described in Jonnalagadda et al. [18]. As described in MILP for finding minimal reaction set, the essential reaction groups (SRGs) are necessary for the cell to meet its cellular objectives and hence these groups have to be present in all minimal reaction sets. Extraneous reaction groups (XRGs) are unnecessary for the cell and will be absent in every minimal reaction set. Indeterminate reaction groups (IRGs) comprise substitutable reactions (see Group substitutability analysis for identifying solutions) which are the source of multiple optimal solutions Hence, all minimal reaction sets can be identified by finding all the different additional reaction groups (ARGs) from the IRGs. These multiple sets of ARGs together with the SRGs identified in Phase 1 forms all the minimal reaction sets.

**Figure 4:**
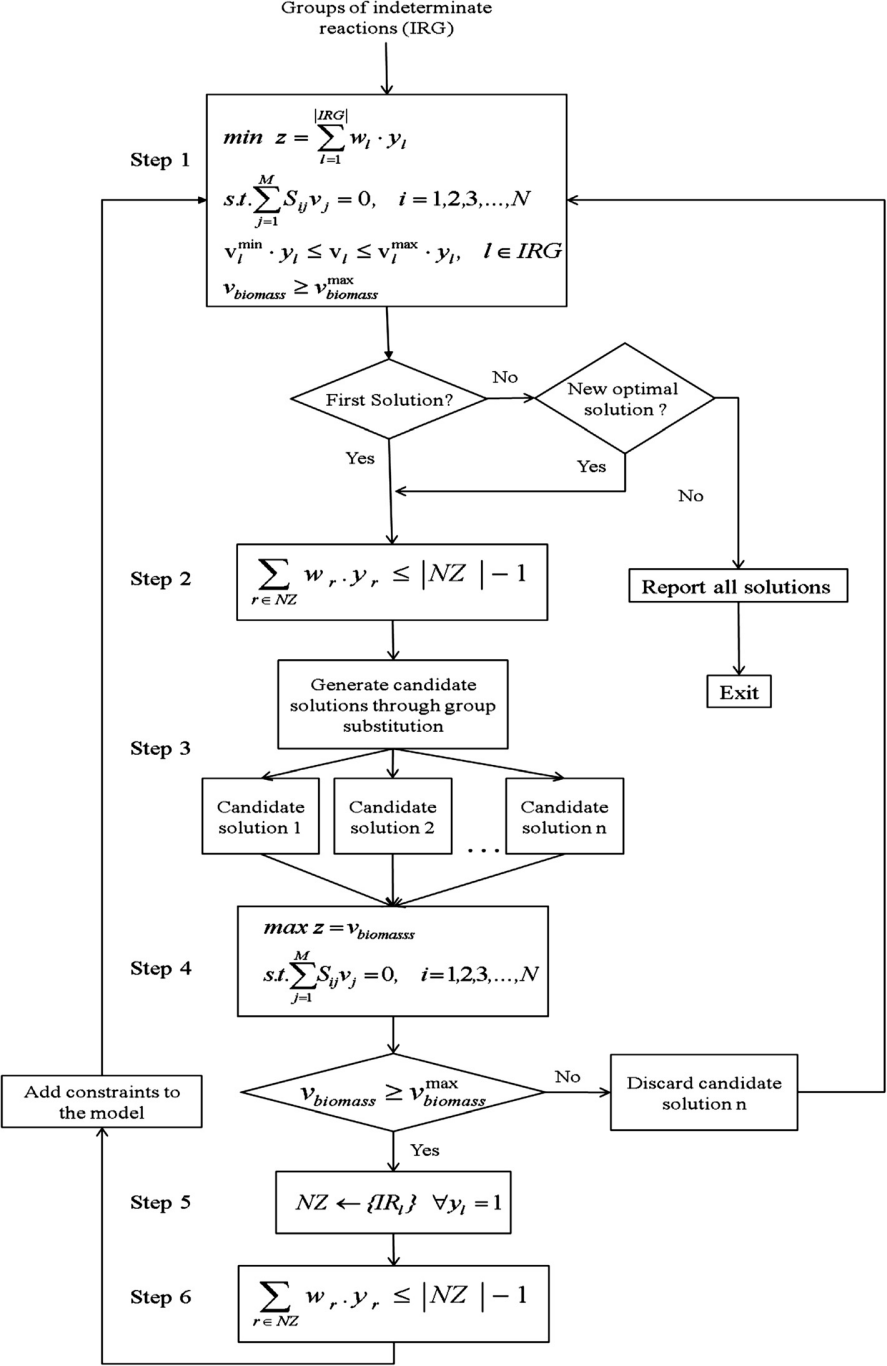
**Recursive MILP approach for identifying all minimal Additional Reaction Groups (ARGs).** After finding an optimal solution, other candidate solutions are generated through substitutability analysis and verified. A new constraint is added to the math program corresponding to each optimal solution, which drives the optimizer to a different optimal solution in the next recursion. The different ARGs identified in Phase 2 together with the Essential Reaction Groups (SRGs) form the various minimal reaction sets.

The algorithm for finding all ARGs from IRGs formulated as a recursive MILP as shown in Figure 4. The first set of ARG is found by solving the MILP with the same constraints as given in Eq. 2, but considering only the IRGs where binary variables have been associated for each group (step 1). The objective function for the optimization is the minimization of $\sum_{l}^{\left|\mathrm{IRG}\right|}w_{l}\cdot y_{l}$ where the *w*_*l*_ is the norm of the group and *y*_*l*_ is the binary variable associated with that group. The optimization procedure thus will identify the ARGs such that the total number of reactions is minimal. The ARGs together with the SRG from Phase 1 forms the first minimal reaction set. Once an optimal solution is found, a constraint is added to the model to exclude that solution from the search space (Step 2). Based on Lee et al. (2000), the following constraint is added to Eq. 2:

(3) $$\sum_{r\in \mathrm{NZ}}w_{r}\cdot y_{r}\leq \left|\mathrm{NZ}\right|-1$$

where *NZ* is the groups in the optimal solution, and *y*_*r*_ is the binary variable associated with the groups in *NZ.* Eq. (3) means that at least one of the non-zero binary variable in the optimal solution is set to zero. Hence in the next recursion, *NZ* is excluded from the search space and the optimizer is forced to find a new optimal solution. This recursive procedure terminates when the optimizer returns a sub-optimal solution, i.e. a solution with more reactions than that in the first solution.

In principle, all the minimal reaction sets can be identified by recursively solving the MILP with a new constraint added to the model in each recursion. However, the grouping of reactions offers insights which enable the computational cost to be reduced significantly by generating additional solutions without solving the MILP, using group substitutability analysis (Step 3).

#### Group substitutability analysis for identifying solutions

In metabolic networks, some reactions share similar cellular functions such as producing, consuming a metabolite, or recycling co-factors. For example, in the sample network shown in Figure 2a reactions *r*_*7*_ and *r*_*8*_ consume metabolite *D* and produce metabolite *G*. The presence of reactions with similar functions enables the cell to survive under different conditions, stress, and malfunction of genes through substitution of reaction for another inactive reaction. These reactions are considered *substitutable* since they result in alternate optima. In the minimal reaction set identification problem, substitutable reactions lead to multiple minimal reaction sets. The above recursive MILP approach can be employed to identify all minimal reaction sets. Alternatively, many candidate solutions can be generated more efficiently by simply substituting a reaction in an optimal solution.

In this paper, we perform this substitution analysis on groups to efficiently identify alternate optimal solutions. Two types of group substitution are possible – single and multi-group substitution. In single group substitution, a group is substituted with another group of the same norm. So, the total number of reactions in the optimal solution remains unchanged. For example, in Figure 2b groups 3 and 4 are substitutable as both have the same metabolic function and have a norm of 1. Thus if group 3 is present in an optimal solution, another candidate solution can be generated by replacing it with group 4. Substitutability for single groups can be identified easily since all the groups would produce and consume the same metabolites, and can hence be identified by OR gates in the graph representation of the metabolic network. Sets of groups could also be analysed for substitutability but this multi-group substitution is computationally complex and is beyond the scope of this paper.

Group substitutability analysis is conducted in the proposed approach following the identification of a solution by the MILP and candidate solutions generated. Not every candidate solution identified by the qualitative approach would meet the cellular objectives. Therefore, it is essential to verify the candidate solutions to ensure that the predefined biological objectives are satisfied. This verification is conducted by solving a linear program (LP) with the objective of maximizing the cellular objective (Step 4), which is computationally efficient. Only candidate solutions that satisfy the objective are deemed as optimal solutions to the original MILP and appended to the set of optimal solutions (Step 5). Other candidate solutions are discarded. A new constraint is also added to the model for each optimal solution thus identified to eliminate their re-identification in future recursions (Step 6). The algorithm then continues with solving the MILP to find other optimal solutions.

## Results

We illustrate the proposed method by identifying all minimal reaction sets that support predefined growth for two systems – *Escherichia coli* and *Saccharomyces cerevisiae*.

### Case Study 1: Aerobic growth of Escherichia coli on glucose

Here, we identify all the minimal reaction sets from the *E. coli* metabolic network so as to meet cellular objective $\nu \begin{array}{c} \max\\ \mathrm{biomass} \end{array}\geq 0.7$ g/gDW∙h for a glucose uptake rate of 10 mmol /gDW∙h. The network contains 63 metabolites and 77 reactions [29]. These 77 reactions are first grouped based on dependency as described in Reaction dependency and grouping. There are in total 62 groups — 3 groups with norm 3, 9 groups with norm 2 and 50 groups with norm 1 *i.e.* single reaction groups. Hence, the number of binary variables required for MILP is reduced from 77 to 62. The proposed recursive MILP method is then employed to identify all the minimal reaction sets from this network. Phase 1 of the proposed approach classified 14 groups (18 reactions) as essential reaction groups of which 4 groups with norm 2 the remaining 10 groups of norm 1. The method also denoted 8 groups (12 reactions) as extraneous. There were 40 groups (47 reactions) identified as indeterminate containing 1 group with norm 3, 5 groups with norm 2 and the remaining 34 with norm 1. Hence, the number of binary variables defined in Phase 2 is reduced from 47 to 40. Then the recursive MILP is employed to identify IRGs. The first optimal solution contains 18 groups (20 reactions) — 17 groups of norm 1 and 1 group with norm 3. The recursive MILP found two more optimal solutions (also with 20 ARs) that meet the predefined cellular objective. In the fourth iteration, the optimizer found a sub-optimal solution with 21 ARS and hence is terminated. These three sets of additional reactions together with the 18 SRs from Phase 1 form the three different minimal reaction sets. To cross-validate the results, we also implemented the classical monolithic MILP approach with 77 binary variables. The monolithic MILP also identified the same three minimal reaction sets thus confirming the accuracy of the proposed approach.

**Figure 5:**
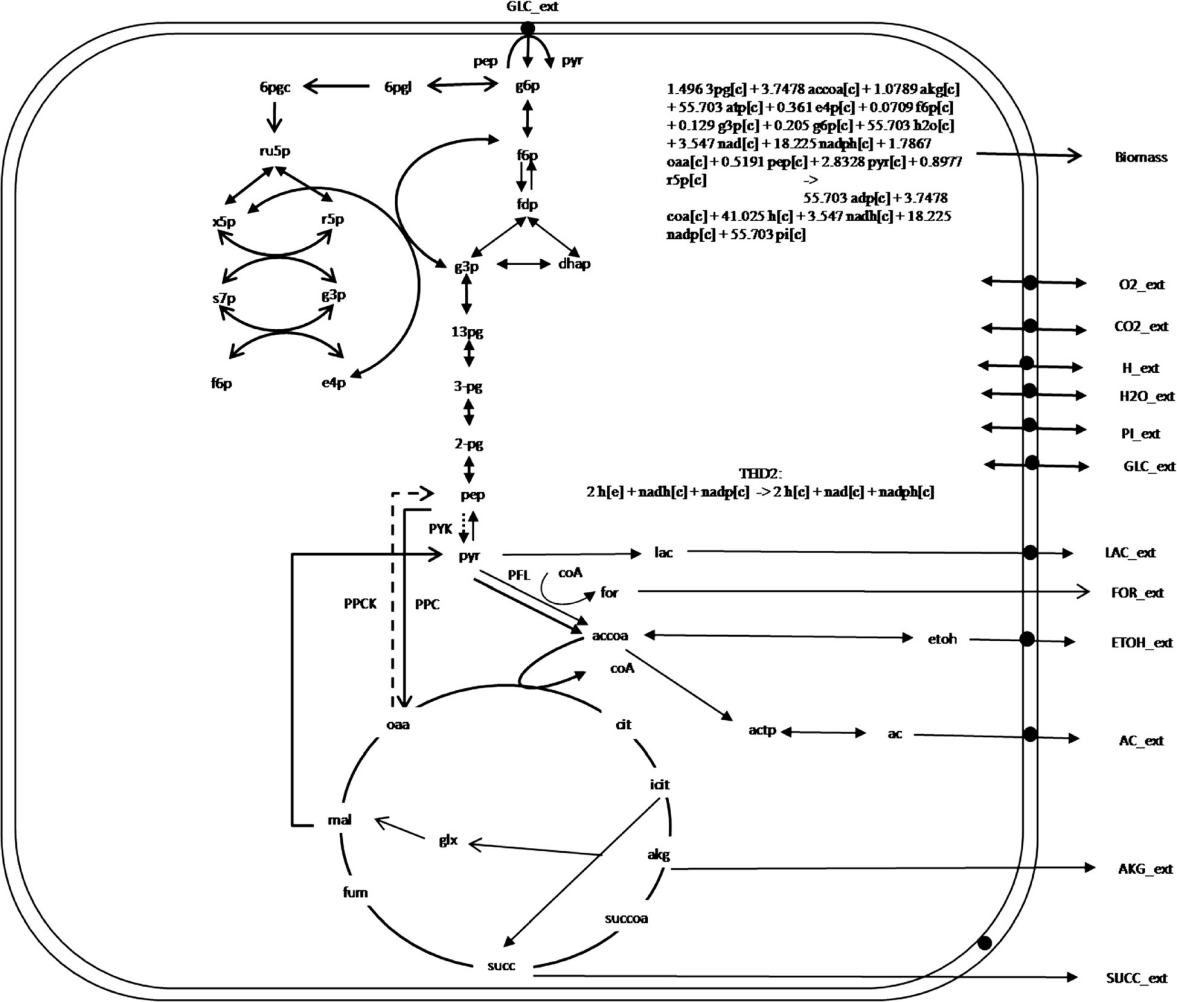
**The metabolic network of *Escherichia coli *used in case study 1.** The reactions in the minimal reaction set are shown by thick solid line. Reactions unique to minimal reaction set 1 (PPCK) is shown as dashed line and the unique reaction in minimal reaction set 2 (PYK) as dotted line. The unique reaction in minimal reaction set 3 (THD2) is a Transhydrogenase reaction involving only cofactors is given as a separate reaction.

The three minimal reaction sets identified in the *E. coli* metabolic network are shown in Figure 5. The reactions in the minimal reaction set are shown by thick solid line. The three minimal reaction sets differ from each other by the presence of a single unique reaction while 37 of 38 reactions in the minimal reaction set are common to all three. This indicates that 19 out of the 20 reactions identified in Phase 2 by recursive MILP are common to all three minimal sets. However, these 19 reactions are deemed as Indeterminate (not as Essential reactions) in Phase 1 since there exist alternative (but sub-optimal) pathways. Minimal reaction Set 1 has a unique reaction Phosphoenolpyruvate carboxykinase (PPCK) while Set 2 has Pyruvate kinase (PYK) and Set 3 has Transhydrogenase (THD2). The comparison of flux distributions from the different reaction sets reveals how the cell meets its biological objective while still staying minimal. For example, minimal reaction Set 1 contains PPCK which converts Oxaloacetate, produced from Phosphoenol pyruvate through Phosphoenolpyruvate corboxylase (PPC) reaction, back to Phosphoenol pyruvate forming a cycle. Since such cycles may not generally be active at steady-state, considering thermodynamics [30], this minimal reaction set may not be suitable for developing minimal metabolism cell. Similarly, minimal reaction set 3 has large flux through the transhydrogenase reaction that regenerates cofactors NADH, NADP from NAD and NADPH. This set is also not desirable for developing minimal metabolism cell since such a high flux may not be practically possible in the organism. In comparison, Set 2 has a unique reaction PYK that converts Phosphoenolpyruvate to Pyruvate which is part of glycolysis pathway in aerobically growing *E.coli* and contains no coupled reactions (cycles); hence, it is a suitable reaction set for developing the minimal metabolism cell.

We identify the number of reactions to be knocked-out from *E.coli* in order to develop minimal metabolism cell based on each minimal reaction set using the graph theory based approach [18]. In brief, the procedure iteratively selects the reaction with lowest depth from the list of reactions not present in the minimal reaction set as the knock-out candidate. In each iteration, all the reactions dependent on the selected reaction are excluded from the list. The procedure continues until the list of reactions becomes empty. Thus all the reactions selected in this procedure have to be removed from the strain to achieve the minimal metabolism based on the selected minimal reaction set. In this case study, all three minimal reaction sets require 6 reactions to be knocked-out from *Escherichia coli*; 4 of these 6 reactions are the same in all cases. Hence, the minimal metabolism cell can be constructed by suitable blocking out the reactions corresponding to minimal reaction set 2. In summary, finding multiple minimal sets enables us to develop the best minimal metabolism cell by selectively deleting the remaining two reactions.

### Case Study 2: Aerobic growth of Saccharomyces cerevisiae on glucose

We now illustrate the computational efficiency of the proposed method by identifying all the minimal reaction sets for a genome-scale model of *Saccharomyces cerevisiae* containing 1061 metabolites and 1266 reactions [31]. The cellular objective is selected as $\nu \begin{array}{c} \max\\ \mathrm{biomass} \end{array}$ of 0.0973 g /gDW∙h for glucose uptake rate of 1 mmol /gDW∙h. The model is reduced to 620 reactions by removing 637 reactions that are not connected to the glycolysis pathway and 9 reactions which differ in a cofactor. There are 114 groups of dependent reactions in the norm range [2 15].

Phase 1 of the proposed approach identified 128 groups (213 reactions) as essential, 22 groups (37 reactions) as extraneous, and 301 groups (370 reactions) as indeterminate. The extraneous reactions are removed from further analysis. Unlike the *E. coli* model, the *Saccharomyces cerevisiae* model has compartments. Out of the 370 indeterminate reactions, 52 reactions are involved in transporting metabolites among the compartments and inter-converting cofactor metabolites. These are deemed to be essential reactions. The remaining 249 groups containing 318 indeterminate reactions are further analyzed in Phase 2 using recursive MILP to find all additional reactions. The results are given in Table 1. There are 38 reactions in the first solution that together with the 265 essential reactions from Phase 1 form the first minimal reaction set with 303 reactions.

**Table 1:** **Results for *Saccharomyces cerevisiae *** **case study**

**Method**	**No. of minimal reaction sets**	**Time required (seconds)**		**Total time (seconds)**
**Monolithic MILP (Jonnalagadda et al. **[18]**)**	**1**	**7200**		**7200**
Graph theory augmented MILP (Jonnalagadda et al. [18])	1	Phase 1	48	1086
		Phase 2 (one solution)	1038	
Proposed approach	256	Phase 1	48	243
		Phase 2 (First Solution)	195	

The proposed approach identified 256 minimal reaction sets within a reasonable amount of time.

Based on the first solution, 7 other minimal reaction sets that meet the predefined cellular objective are identified through group substitutability analysis leading to a total of 8 minimal reaction sets in the first iteration. These 8 optimal solutions were excluded from the search space through addition of new constraints. In the seconds iteration, 6 more minimal reaction sets were identified — 1 from MILP and 5 from substitution analysis. The algorithm then continues with next iteration. The results for each iteration are shown in Table 2. There are a total of 256 minimal reaction sets for this metabolic network. The proposed recursive MILP approach has to go through 66 iterations to identify all these optimal solutions. Further execution of MILP resulted in a sub-optimal solution with 39 reactions, hence it terminated.

**Table 2:** **Results for *Saccharomyces cerevisiae *** **case study for all the iterations in Phase 2**

**Run**	**Time taken (sec)**	**CPLEX iterations**	**Time taken for group substitutability analysis (sec)**	**No of optimal solutions identified by substitutability analysis**	**Total no. of solutions**
1	194.24	1,557,244	0.0163	7	8
2	169.57	1,324,661	0.0144	5	14
3	375.11	3,156,253	0.0156	7	22
4	326.91	2,825,007	0.0145	3	26
5	329.70	2,600,338	0.0158	6	33
6	499.10	3,999,565	0.0143	5	39
7	395.26	3,097,534	0.0146	1	41
8	346.00	2,604,183	0.0151	5	47
9	443.81	3,227,776	0.0141	3	51
10	341.17	2,919,954	0.0147	5	57
11	605.92	3,605,713	0.0152	5	63
12	469.40	3,628,574	0.0152	6	70
13	462.00	3,709,793	0.0154	5	76
14	499.12	3,957,554	0.0154	5	82
15	493.20	3,705,021	0.0158	1	84
16	508.61	3,541,896	0.0139	3	88
17	461.61	3,717,958	0.0156	7	96
18	586.31	3,896,863	0.0147	2	99
19	572.73	3,834,966	0.015	2	102
20	101.44	727,992	0.0147	5	108
21	70.72	559,520	0.0148	3	112
22	87.30	666,870	0.0147	5	118
23	83.13	626,335	0.0152	2	121
24	80.39	622,837	0.0152	1	123
25	89.23	650,657	0.0149	1	125
26	143.5	909,324	0.0145	5	131
27	96.71	702,949	0.015	6	138
28	94.68	672,714	0.0146	3	142
29	155.29	939,499	0.0151	1	144
30	95.20	669,098	0.0154	1	146
31	167.19	939,535	0.0145	1	148
32	96.33	691,134	0.0145	3	152
33	167.44	945,667	0.0148	2	155
34	136.38	814,707	0.0153	0	156
35	197.90	1,104,430	0.0147	2	159
36	98.23	668,304	0.0151	2	162
37	191.12	978,923	0.0149	5	168
38	110.01	745,564	0.0154	7	176
39	198.40	987,311	0.015	6	183
40	119.95	755,954	0.0149	3	187
41	190.69	986,746	0.015	5	193
42	115.18	723,329	0.0152	0	194
43	105.69	675,581	0.0153	2	197
44	334.70	1,065,005	0.0149	6	204
45	105.12	675,556	0.0158	7	212
46	262.07	1,073,140	0.0155	0	213
47	135.00	813,478	0.0152	1	215
48	200.65	957,317	0.0147	0	216
49	215.34	978,111	0.0152	5	222
50	251.66	1,209,539	0.0154	6	229
51	196.72	1,016,461	0.0151	1	231
52	263.74	1,289,731	0.0153	0	232
53	270.45	1,284,578	0.0147	0	233
54	131.65	770,203	0.0153	5	239
55	227.39	1,052,628	0.0152	1	241
56	255.51	1,156,613	0.0156	1	243
57	131.90	770,250	0.0155	1	245
58	142.50	837,089	0.0151	0	246
59	367.53	1,775,515	0.0151	0	247
60	317.67	1,553,221	0.0157	1	249
61	223.43	1,106,047	0.0149	0	250
62	178.30	1,042,046	0.0156	1	252
63	342.01	1,553,461	0.0153	0	253
64	170.40	920,427	0.0148	0	254
65	260.28	1,237,089	0.015	0	255
66	206.23	1,125,379	0.0161	0	256
Total	16262.53	104,938,717	0.9953		256

To quantify the improvement achieved, we executed the MILP with 318 binary variables for 318 indeterminate reactions. The solver required 3,735,864 CPLEX iterations and 1038 seconds to find the optimal solution. The reduction of number of binary variables has resulted in a significant improvement with approximately 60% reduction in CPLEX iterations and 80% reduction in the time required for finding the optimal solution in Phase 2. We also compared the total time required for Phase 1 & 2 to find the first solution by the proposed method with monolithic MILP and graph theory based approach without grouping. The results are given in Table 1. The time required by the proposed method is ~ 4% and 22% of the time required for the monolithic MILP and graph theory based approach, accordingly. For all 256 solutions, the proposed approach required 16311 seconds. The large computational time required for monolithic MILP restrained its use for finding all minimal reaction sets. Nonetheless, to validate the results monolithic MILP was employed after excluding all the 256 minimal reactions from search space. It found a sub-optimal solution with 304 reactions in the minimal set. This guarantees that the proposed method identifies all minimal reaction sets.

## Discussion and conclusions

Development of cells with minimal metabolic functionality is increasingly gaining importance. The presence of redundant reactions in metabolic networks results in multiple minimal reactions sets that can meet the predefined cellular objectives. In this paper, we proposed a graph theory augmented recursive MILP approach to identify all the minimal reaction sets in a metabolic network. The proposed method has been demonstrated by finding all the minimal reaction sets for *Escherichia coli* and *Saccharomyces cerevisiae*. The proposed approach correctly identified all the minimal reaction sets in both the cases. We also proposed the concept of grouping dependent reactions to reduce the number of binary variables for MILP formulation. In the present study, several groups of dependent reactions are identified in *Escherichia coli* and *Saccharomyces cerevisiae* and exploited to reduce the number of binary variables and consequently the solution time. Since the use of binary variables is very common in metabolic network analysis for identifying strain improvement strategies [24,25,27], the reaction group concept will benefit the other applications as well.

Here, we have developed a graph based algorithm that exploits the structure of the metabolic network to identify groups of dependent reactions. We now compare the groups of dependent reactions identified by the proposed method with the previously reported approach based on steady-state flux distribution. We used the METATOOL software [32] to find the dependent reaction groups using steady-state flux distribution. While the proposed graph based approach found 114 reaction groups (containing 291 unique reactions) with norm more than 1 in the yeast model used in case study 2, the flux based approach identified 86 dependent reaction groups (with 277 unique reactions). The norm range of the reaction groups identified by the proposed approach is [2 15] while that for the flux based approach is [2 21]. The lower range of the proposed approach could be due to the strict use of the structure of the metabolic network in contrast to the flux based approach which can place structurally unconnected reactions in a group. Nonetheless, the use of structure of metabolic network identified some dependent reaction groups that were not identified by the flux based approach due to the use of reaction directionality as described in Reaction dependency and grouping. While there is a large overlap between the dependent reaction groups found by the two approaches, several groups are uniquely identified by each approach. Hence, combining the two approaches could further simplify the metabolic network.

Generally, different minimal reaction sets have different structural and functional properties in terms of robustness, predictable metabolic interactions, practically achievable metabolic fluxes, and thermodynamically non-favourable pathways and cycles. We identified one such cycle in one of the solutions identified in *E.coli* model in case study 1 and very high flux through co-factor recycling reaction in another solution. Thus, of the three, only one solution has practically achievable fluxes without any coupled reactions (cycles) and is suitable for developing the minimal metabolism cell. This clearly shows the importance of identifying all minimal reaction sets. In Case Study 2, the proposed method identified 256 solutions. While some of them use glycolysis pathway to produce DHAP others use pentose phosphate pathway. Other differences observed include use of different reactions for the consumption of DHAP and formate, recycling of NAD, NADH, NADP, and NADPH, and production of Ammonium. A preliminary analysis shows that some minimal reaction sets require complex set of reactions with impractical high fluxes compared to others. Further analysis may reveal the details of these pathways that qualify them to developing into minimal metabolism cells.

## Competing interests

The authors declare that they have no competing interests.

## Authors’ contributions

Both SJ and RS contributed to the concept and methodology development. SJ implemented the methodology and conducted the analysis of results and biological interpretation. RS supervised the study and assisted in implementation. SJ drafted the manuscript. Both authors read and approved the final manuscript.
